# Supplementary material for: Decadal decrease in Los Angeles methane emissions is much smaller than bottom-up estimates
Source: Nat Commun. 2023 Sep 2;14:5353. doi: 10.1038/s41467-023-40964-w (PMC10475107; doi:10.1038/s41467-023-40964-w)
Supplement: Supplementary file 1 — Supplementary Information [file 41467_2023_40964_MOESM1_ESM.pdf]

Supplementary Information for

Decadal decrease in Los Angeles methane emissions is much smaller than bottom-up estimates

Zhao-Cheng Zeng[1,*], Thomas Pongetti[2], Sally Newman[1,3], Tomohiro Oda[4,5,6], Kevin Gurney[7], Paul I. Palmer[8], Yuk L. Yung[1,2], and Stanley P. Sander[2,1,*]

[1] Geological and Planetary Sciences, California Institute of Technology, Pasadena, CA, US

[2] Jet Propulsion Laboratory, California Institute of Technology, Pasadena, CA, US

[3] Present address: Planning and Climate Protection Division, Bay Area Air Quality Management District, San Francisco, CA, US

[4] Earth from Space Institute, Universities Space Research Association (USRA), Columbia, MD, US

[5] Department of Atmospheric and Oceanic Science, University of Maryland, College Park, MD, US

[6] Graduate School of Engineering, Osaka University, Suita, Osaka, Japan

[7] School of Informatics, Computing, and Cyber Systems, Northern Arizona University, Flagstaff, AZ, US

[8] School of GeoSciences, University of Edinburgh, Edinburgh, UK

* Corresponding authors: S. P. Sander (stanley.p.sander@jpl.nasa.gov) and Z.-C. Zeng (zcz@gps.caltech.edu)

Contents of this file

Supplementary Text 1 to Supplementary Text 3
Supplementary Figure S1 to Supplementary Figure S15

Supplementary Text 1: Deriving CH_4/CO_2 excess ratio from NOAA flask data

To verify the seasonal cycle of CH_4/CO_2 ratio, we calculate the excess ratio using the NOAA MWO daytime and nighttime flask data (**Supplementary Figure S4**) and compare with estimates using CLARS-FTS data. Similar to **Hsu et al.**[1], we used the corresponding nighttime flask measurements on the same day as the background and subtracted the backgrounds from the daytime measurements to get the CO_2 excess ($CO_{2,xs}$) and CH_4 excess ($CH_{4,xs}$). Their correlation and the monthly means of the excess ratios are shown in **Supplementary Figure S5**.

Supplementary Text 2: Background estimation for CLARS-FTS

The details of the background calibration are introduced in **He et al.**[2]. To derive an unbiased background XCH_4 and XCO_2 along the same path of the CLARS target mode, we combined the CLARS Spectralon retrievals and NOAA in situ flask dataset at Mt. Wilson (https://gml.noaa.gov/dv/site/site.php?code=MWO). The NOAA in situ flask dataset gives the background estimate using in situ flask-based sampling at Mt. Wilson next to the CLARS facility. At night, the height of the boundary layer falls to far below the CLARS facility and the flask record is very likely to represent background conditions for the lower troposphere over the region, where there are no human activities. Therefore, to construct the background, we used the Spectralon measurements as the background for the atmosphere above the CLARS height, and the NOAA flask measurements at night as the background for the atmosphere below. **Supplementary Figure 1** shows a comparison of the time series of CLARS observations for the surface target mode and the Spectralon mode, and the NOAA nighttime flask monthly averaged data.

Supplementary Text 3: The contribution of biogenic fluxes to CO_2 enhancement in LA

Newman et al.[3] used $^{14}CO_2$ flask data to better constrain contributions from anthropogenic emissions and the biosphere to the observed CO_2 enhancement in the Los Angeles Basin. They found that the largest biospheric contribution occurred during winter 2012–2013 (7 ppm - 28 % of the total C_{ff}, where C_{ff} is the CO_2 contribution from fossil fuel combustion), and the minimum was 0.1 ppm during spring of 2010. The average is (4.1 ± 0.5) ppm (16 % of C_{ff}) during cooler months and (2.2 ± 0.3) ppm (8 % of C_{ff}) during warmer months. An extension of the time series is shown in **Supplementary Figure S6**. In **Miller et al.[4]**, which used measurements of $\Delta^{14}C$ and CO_2 to separate biogenic and fossil contributions to CO_2 enhancements above background, found that the urban biospheric component is a source in winter and a sink in summer, with an estimated amplitude of 4.3 parts per million (ppm), equivalent to 33% of the observed annual mean fossil fuel contribution of 13 ppm. The $CO_{2,xs}/CO_{2,ff}$ -1.0 monthly mean data are shown in **Supplementary Figure S7**. In addition, we examine the impact on the inter-annual trend. **Supplementary Figure S6**, extended from the results in **Newman et al.[3]**, shows biogenic contribution from 2006 and mid-2016 (data after 2017 are not available). The slope of the CO_2bio trend is 0.013 ppm/year, which is very small and indicates no significant trend. Based on this result, it is assumed the biogenic fluxes in the basin do not have a significant interannual trend that could affect the derived methane emissions in the paper.

Supplementary Figure S1. Time series of CLARS-FTS observations for the surface target mode and the Spectralon mode, and the NOAA nighttime flask monthly averaged data.

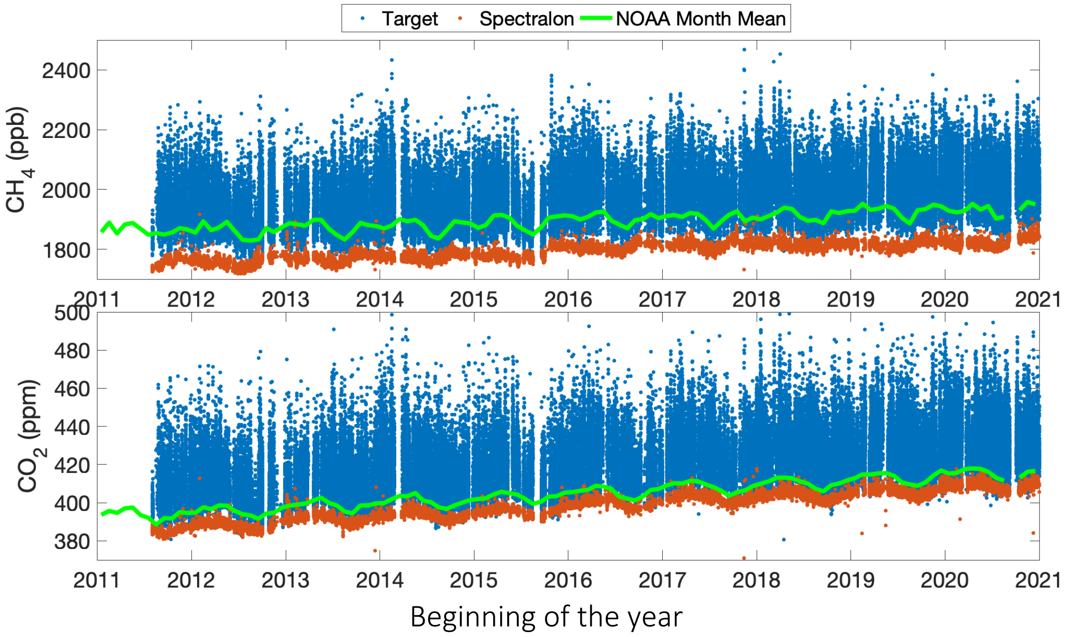

Supplementary Figure S2. A comparison of CO_2 bottom-up emission inventories from Hestia (red; 2011-2015), CARB (green; 2011-2019), and ODIAC (blue; 2011-2019). CARB inventory values for the LA basin are scaled from the state's total emissions using the ratio of SOCAB population (14.6 million) to total California population (39 million). ODIAC is shifted upward by 3.5 $TgCO_2$/month to match the Hestia annual estimate. After adjusting to the Hestia level, 10% uncertainty is assumed for these monthly estimates (similar to the uncertainty estimate of Hestia by **Gurney et al.**[5]). For CO_2 emissions in 2020, we used the 2019 value as the baseline and applied scale factors from **Yadav et al.**[6] to derive the drawdown of CO_2 emissions in LA due to the COVID-19 pandemic lockdown. This extrapolation is applied to ODIAC and CARB inventories. See text for details.

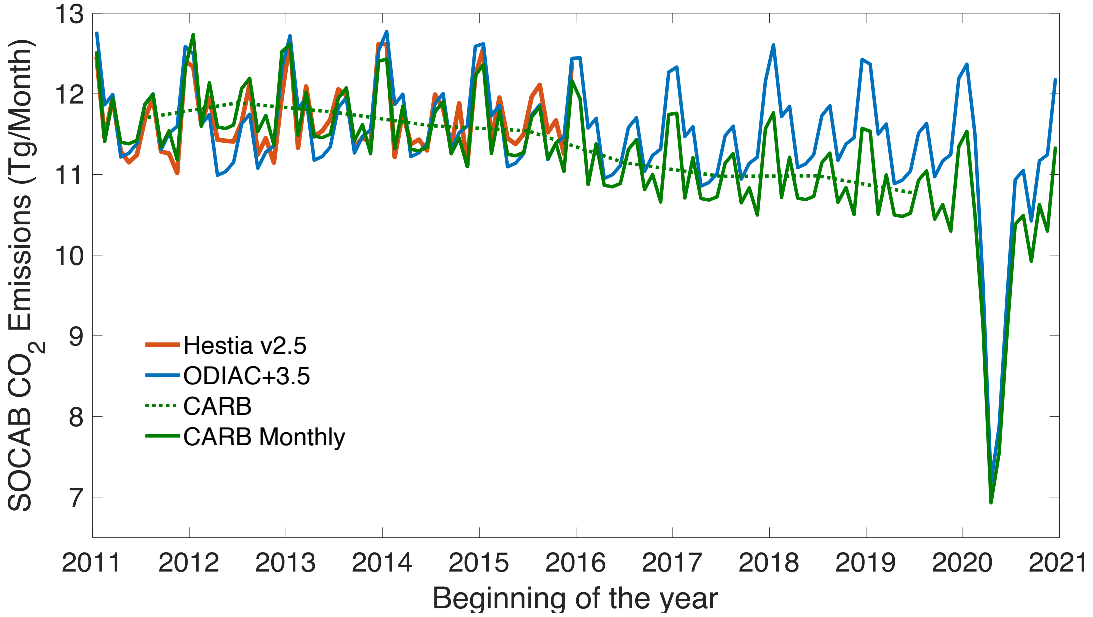

Supplementary Figure S3. 2-D histogram of XCO_2,xs and XCH_4,xs from CLARS-FTS observations. The color bar indicates the observation density, which is the number of measurement pairs. From the linear regression, as shown in the red dashed line, the intercept = 5.75 ppb and the slope = 6.38 ppb/ppm.

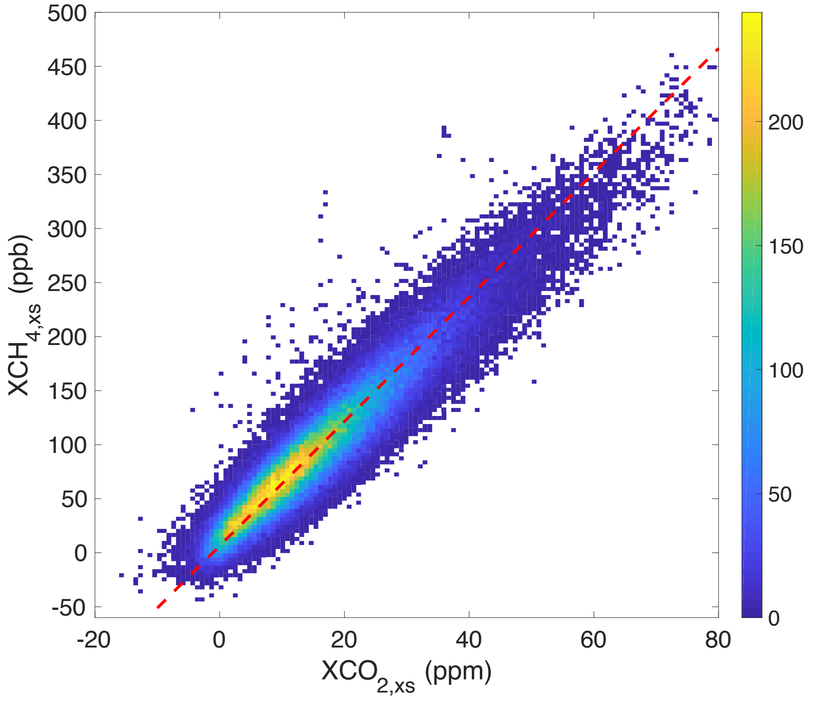

Supplementary Figure S4. NOAA in-situ nighttime and daytime flask measurements on Mt. Wilson for CH_4 and CO_2.

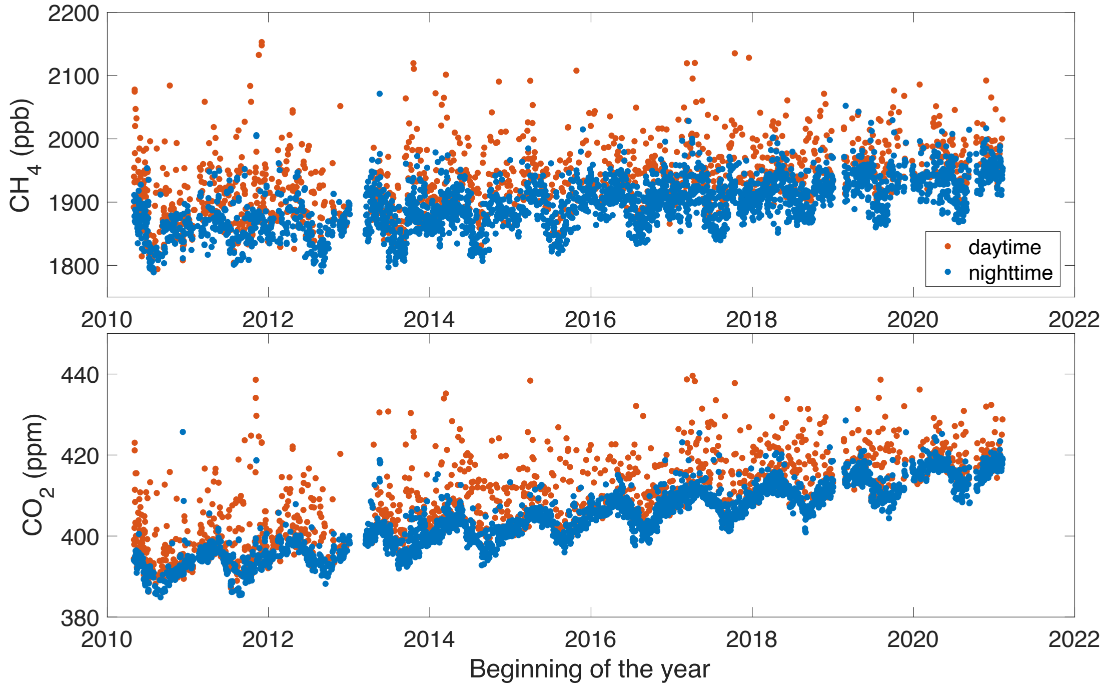

Supplementary Figure S5. (a) scatter plot between CO_2 excess and CH_4 excess from NOAA MWO flask measurements from 2011 to 2020. Only data with positive excesses are used. The correlation coefficient is 0.82; (b) The monthly mean (in red) of excess ratio from NOAA MWO flask measurements from 2011 to 2020. The error bars represent the estimation uncertainty (one standard error) of the monthly values. We filtered the data by using CO_2 excess larger than 5 ppm to exclude days with air not transported from the LA basin.

(a) (b)

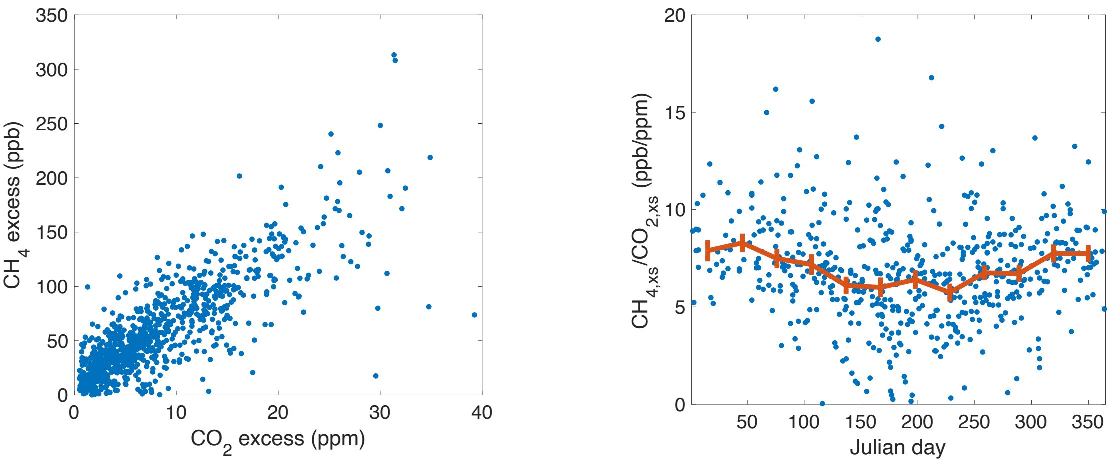

Supplementary Figure S6. The contribution from biosphere and fossil fuel constrained from $^{14}CO_2$ data, a time series extended from **Newman et al.**[3]. The slope of the $CO_{2,bio}$ trend is 0.013 ppm/year from linear regression analysis.

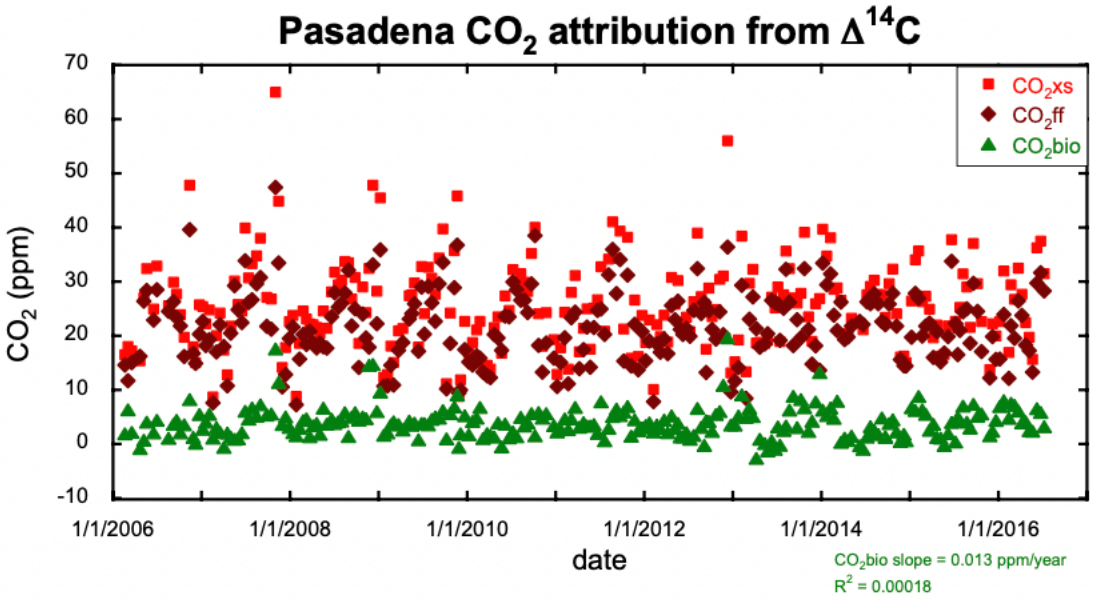

Supplementary Figure S7. Monthly mean of (C_{xs}/C_{ff} – 1.0) inferred from $^{14}CO_2$ data by an extended timeseries from **Newman et al.**[3], as shown in **Supplementary Figure S6**, and **Miller et al.**[4], respectively. The error bars represent the estimation uncertainty (1σ) of the monthly values.

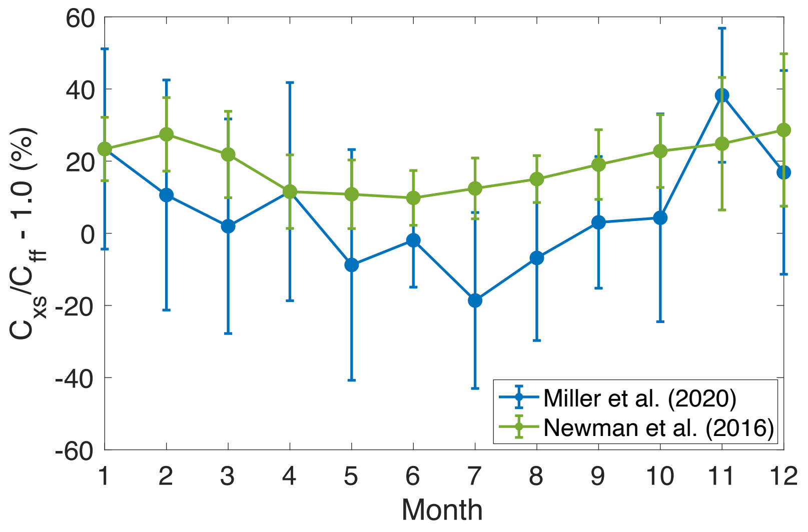

Supplementary Figure S8. Excess ratio of XCH_4,xs/XCO_2,xs before and after correcting the biogenic fluxes derived by an extended timeseries from **Newman et al.**[3], as shown in **Supplementary Figure S6**, and **Miller et al.**[4], respectively. By fitting using a statistical model (**Equation (4)**) that consists of a linear component and a seasonal component by harmonic functions. The slopes of the linear components are -0.022±0.028 (ppb/ppm)/month and -0.026±0.026 (ppb/ppm)/month, respectively, for the corrected timeseries based on **Newman et al.**[3] and **Miller et al.**[4]. The error bars represent the estimation uncertainty (1σ) of the monthly values.

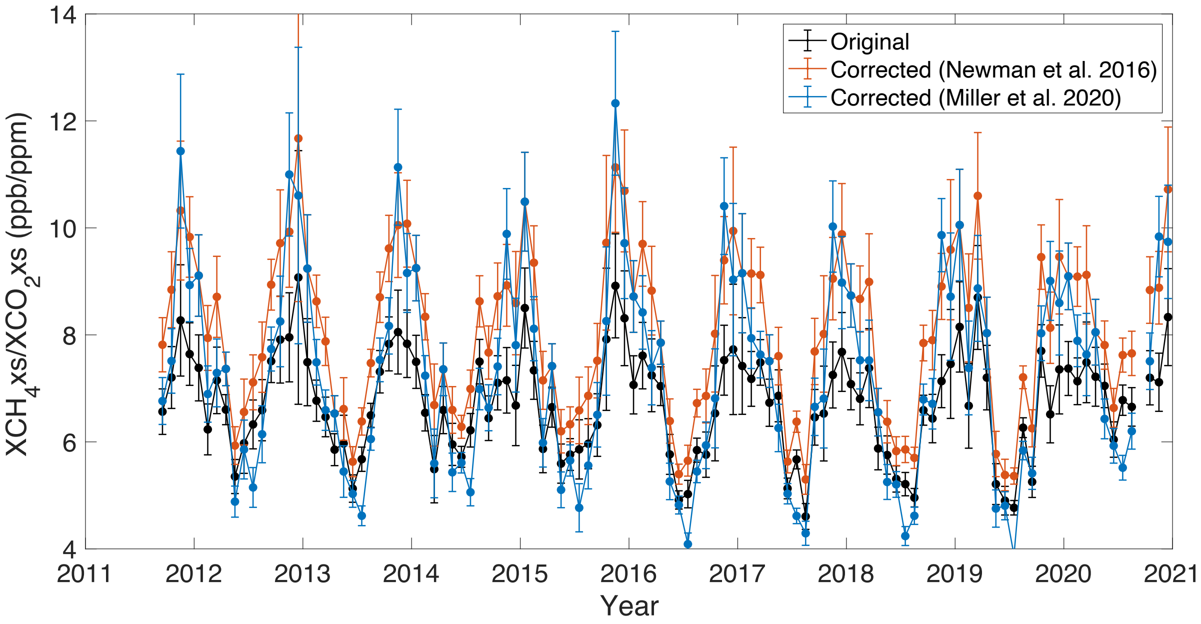

Supplementary Figure S9. Correlation between natural gas consumption and CH_4 emissions estimated using the (a) ODIAC and (b) CARB CO_2 bottom-up inventories, respectively, and biogenic flux correction based on **Miller et al.[4]**. The error bars represent the estimation uncertainty (1σ) of the monthly values.

(a) ODIAC (b) CARB

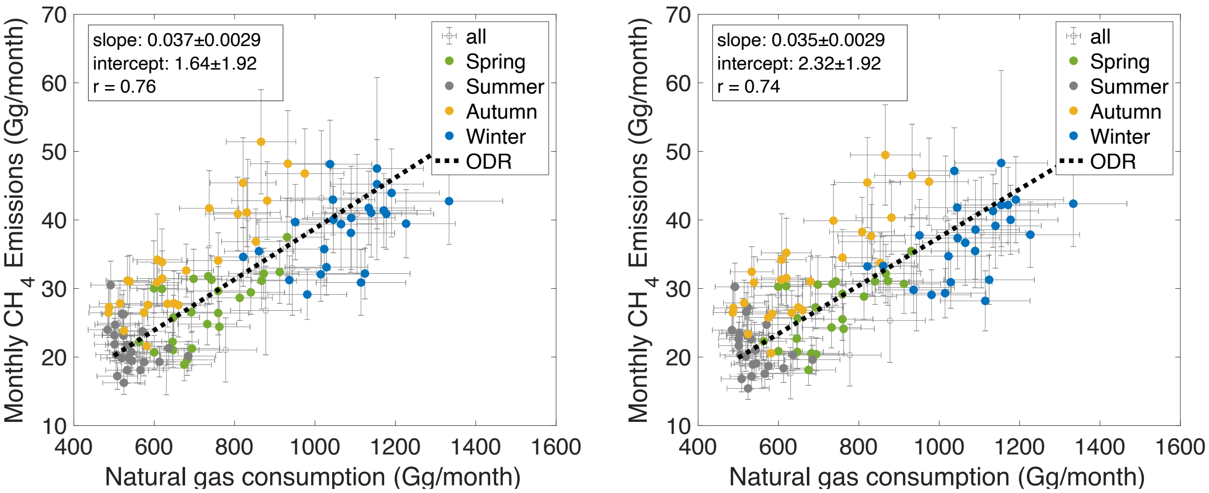

Supplementary Figure S10. Similar to **Figure 3(a)** and **(b)** but for CH_4 emissions estimated using biogenic flux correction based on **Miller et al.[4]**. The error bars represent the estimation uncertainty (1σ) of the monthly values.

(a) ODIAC

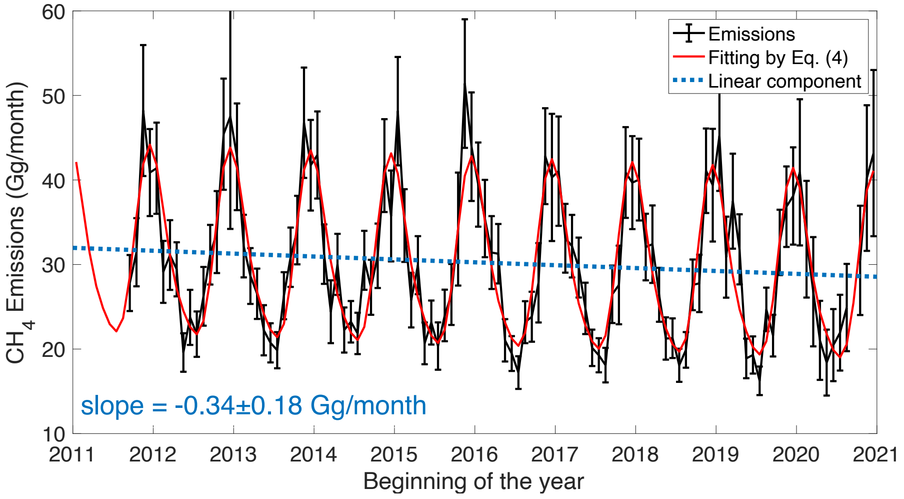

(b) CARB

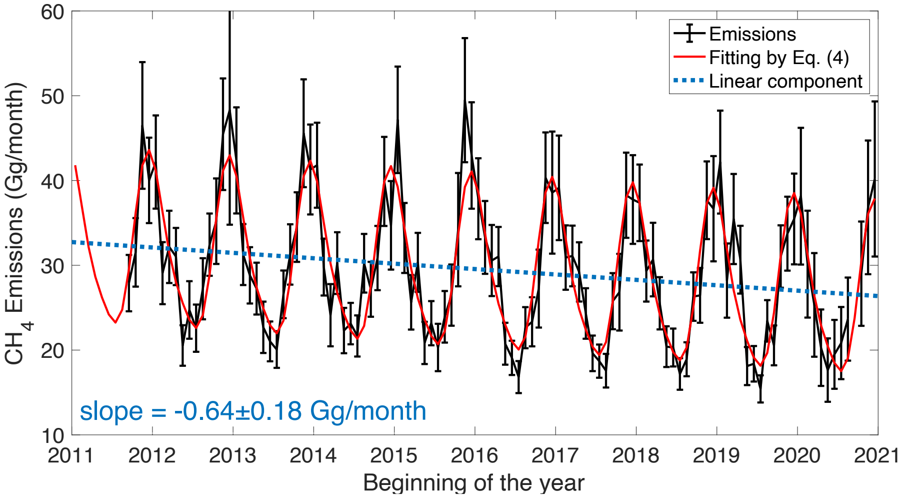

Supplementary Figure S11. Monthly averaged CH_4 emissions for period (1) from January to December, (2) from July to December, and (3) from January to June over 2011 to 2020. The slopes from linear regression over 2011-2019 are -0.41±0.22 Gg/year, -1.03±0.34 Gg/year, and -0.20±0.28 Gg/year, respectively. Note that the year 2020 is not used in the linear regression. The uncertainties of the slopes are estimated using the Monto Carlo method as described in **Methods**. The error bars represent the estimation uncertainty (1σ) of the monthly values.

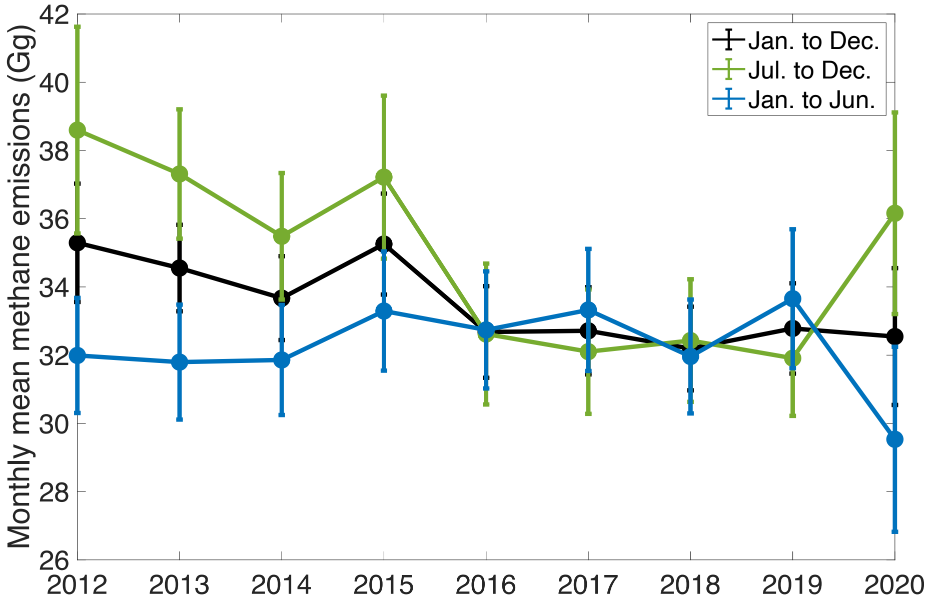

Supplementary Figure S12. Histograms of slopes from Monte Carlo simulations for uncertainty estimations of the emissions trend as shown in **Figure 3** of the main text. The uncertainties for the slope in each case are estimated using the Monte Carlo method, which samples the monthly emissions using a normal distribution based on the mean and error and estimates of the slope. The method makes 10,000 simulations for the emissions time series and obtains the standard deviation of the slope samplings.

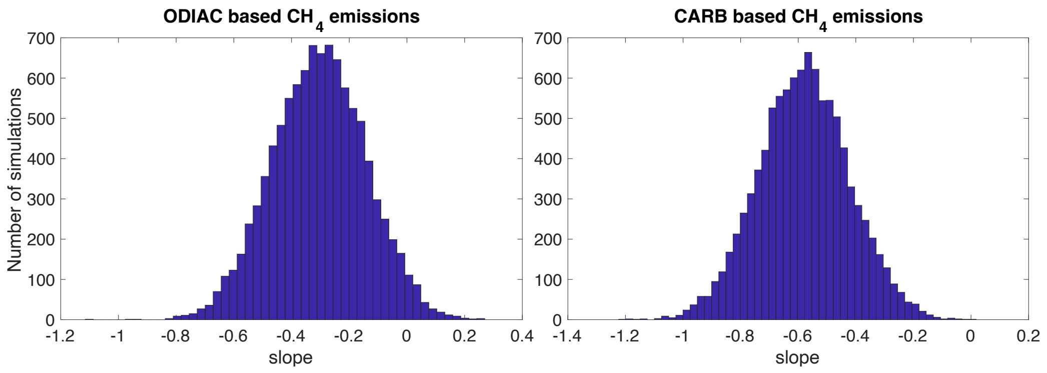

Supplementary Figure S13. The time series of $XCH_{4,xs}/XCO_{2,xs}$ ratio averaged from the three sub-regions in the basin, including western, central and eastern regions of the Los Angeles Basin. The background image in the upper panel is adopted from the Map data ©2019 Google. The error bars represent the estimation uncertainty (1σ) of the monthly values.

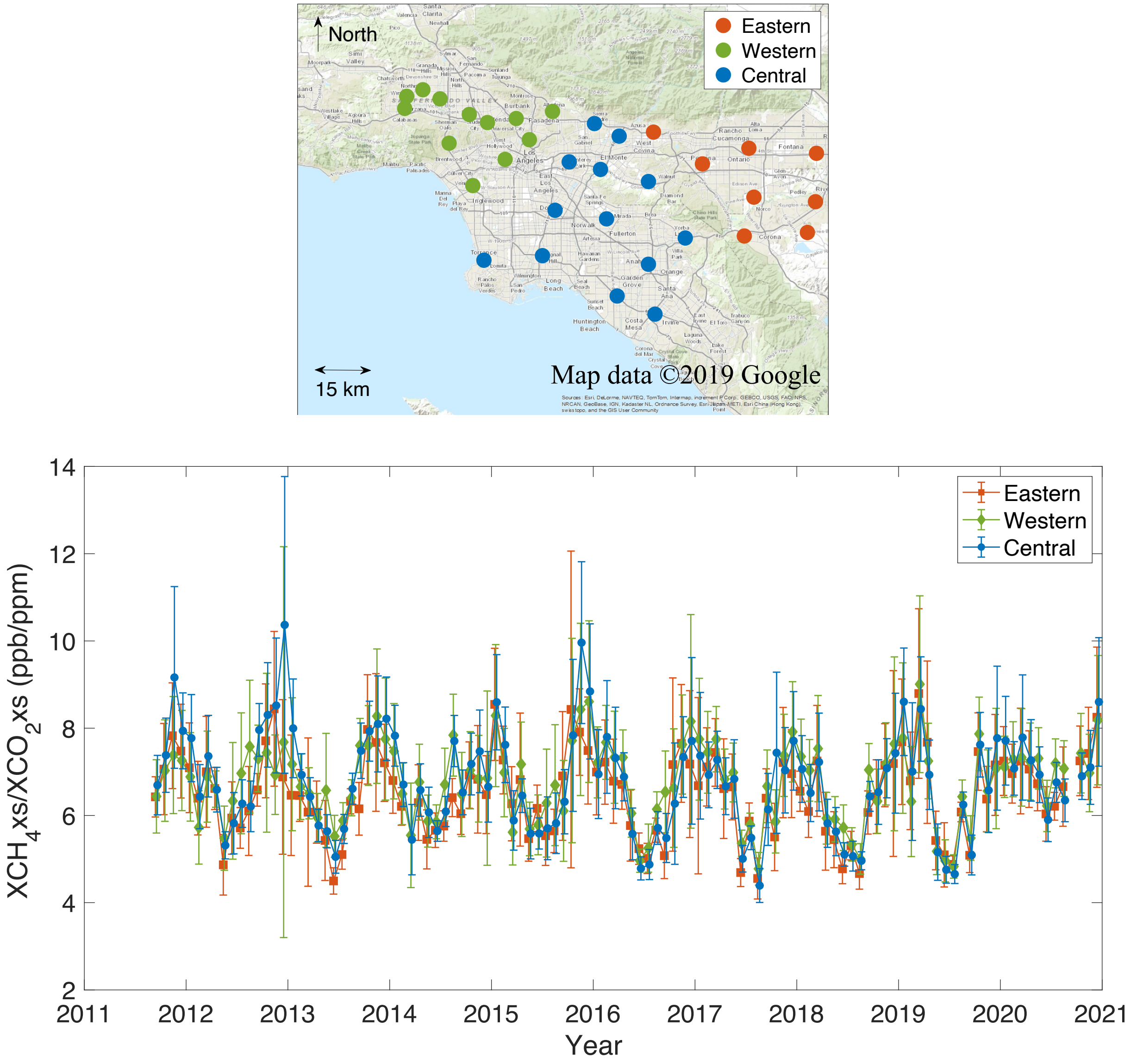

Supplementary Figure S14. Results from EEMD analysis for the CH_4 emissions shown in Figure 2(a), which is estimated using biogenic flux correction from **Newman et al.[3]** and ODIAC CO_2 bottom-up inventories. The EEMD results include (2nd panel) IMF1, (3rd panel) the combination of IMF2 and IMF3, and (4th panel) the combination of IMF4, IMF5, and the trend. The trend in the 4th panel before 2012 and after 2019 is due to the edge effect that can be ignored. The uncertainty band is ±1σ.

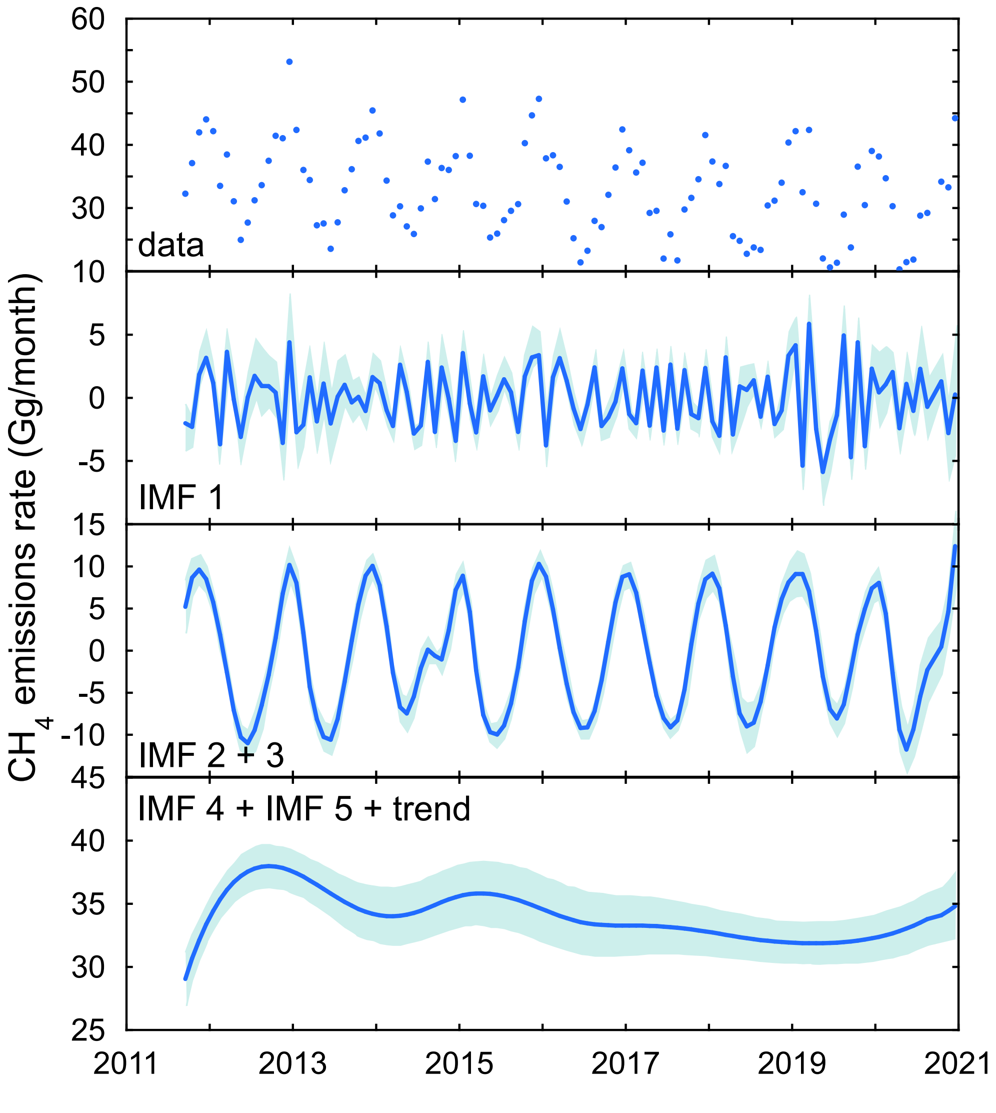

Supplementary Figure S15. The same as **Supplementary Figure S14,** but for the CH_4 emissions estimated using biogenic flux correction from **Newman et al.[3]** and CARB CO_2 bottom-up inventories. The uncertainty band is ±1σ.

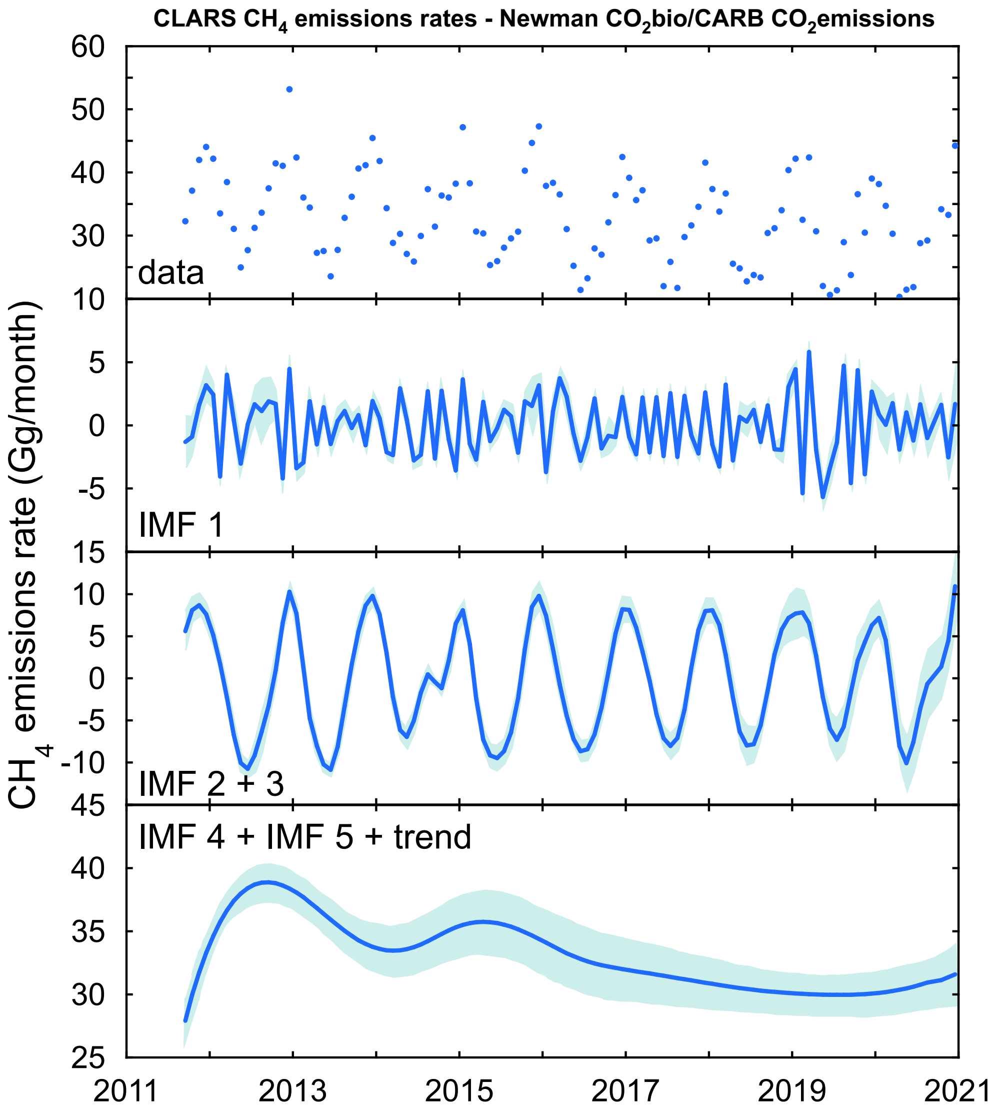

Supplementary References

1. Hsu, Y. K. et al. Methane emissions inventory verification in southern California. *Atmospheric Environment*, *44*(1), pp.1-7 (2010).
2. He, L. et al. Atmospheric methane emissions correlate with natural gas consumption from residential and commercial sectors in Los Angeles, *Geophysical Research Letters*, 46, 8563–8571, https://doi.org/10.1029/2019GL083400 (2019).
3. Newman, S. et al. Toward consistency between trends in bottom-up CO_2 emissions and top-down atmospheric measurements in the Los Angeles megacity, *Atmospheric Chemistry and Physics*, 16, 3843–3863, https://doi.org/10.5194/acp-16-3843-2016 (2016).
4. Miller, J. B. et al., Large and seasonally varying biospheric CO_2 fluxes in the Los Angeles megacity revealed by atmospheric radiocarbon, *Proceedings of the National Academy of Sciences*, 117(43), 26681–26687, https://doi.org/10.1073/pnas.2005253117 (2020).
5. Gurney, K. R. et al. The Hestia fossil fuel CO_2 emissions data product for the Los Angeles megacity (Hestia-LA), *Earth System Science Data*, 11, 1309–1335, https://doi.org/10.5194/essd-11-1309-2019 (2019).
6. Yadav, V. et al. The impact of COVID-19 on CO_2 emissions in the Los Angeles and Washington DC/Baltimore metropolitan areas. *Geophysical Research Letters*, 48, e2021GL092744. https://doi.org/10.1029/2021GL092744 (2021).
